# Social Transfers for Exclusive Breastfeeding in Brazil: Protocol for a Randomized Controlled Trial

**DOI:** 10.2196/75796

**Published:** 2025-09-24

**Authors:** Stephanie Khoury, Alexandra Brentani, Helena Brentani, Jarlei Fiamoncini, Rossana Francisco, Ana Carolina Onofre, Silvia Elise Rodrigues Henrique, Günther Fink, Jordyn Wallenborn

**Affiliations:** 1 Swiss Tropical and Public Health Institute Allschwil Switzerland; 2 University of Basel Basel Switzerland; 3 Department of Pediatrics, Child Development Center – CEDI Faculty of Medicine Universidade de São Paulo São Paulo Brazil; 4 Insitute of Psychiatry Faculty of Medicine Universidade de São Paulo São Paulo Brazil; 5 Department of Food Science and Experimental Nutrition Faculty of Medicine Universidade de São Paulo São Paulo Brazil; 6 Department of Obsterics and Gynecology Faculty of Medicine Universidade de São Paulo São Paulo Brazil

**Keywords:** breastfeeding, lactation, human milk, social transfer, child, mother, Brazil, health

## Abstract

**Background:**

According to the World Health Organization’s infant and young child feeding guidelines, infants should be exclusively breastfed for the first 6 months of life. Despite public health campaigns to increase exclusive breastfeeding (EBF) rates, socioeconomic inequities persist among low-income breastfeeding mothers, especially in countries with large wealth and health gaps, such as Brazil. Social transfer programs are initiatives that provide financial support to individuals or households to improve their well-being and reduce financial burdens. These may be conditional, requiring recipients to meet specific criteria to receive the transfer, or unconditional, in which recipients receive the transfer without prerequisites. Evidence suggests that conditional and unconditional social transfers may help increase EBF rates while addressing the economic challenges breastfeeding mothers face. A randomized controlled trial (RCT) conducted in Vientiane, Lao People’s Democratic Republic, found that a social transfer program significantly improved both EBF rates at 6 months and EBF duration. Building on this study, we aim to evaluate the impact of this intervention in a different socioeconomic and cultural context.

**Objective:**

This protocol aims to implement an RCT to assess whether conditional and unconditional social transfers improve EBF rates at 6 months postpartum for mothers in low-income communities in São Paulo, Brazil.

**Methods:**

A prospective RCT will be conducted among 400 mothers who gave birth in the last 72 hours and plan to exclusively breastfeed. Participants will be recruited in São Paulo at the University Hospital of São Paulo and Amparo Maternal. Participants will be randomly assigned to one of the following groups: (1) control group—no social transfer; (2) intervention group 1—an unconditional social transfer at 6 months postpartum; and (3) intervention group 2—a social transfer at 6 months postpartum, conditional upon mothers’ EBF. All groups will receive educational materials supporting EBF. The study will have visits at birth, 1 month, 6 months, 1 year, and 2 years and will include a questionnaire and biological collections of breast milk samples, infant fecal samples, and blood samples (finger pricks) from both the mother and infant. The main study outcomes are the prevalence of EBF at 6 months and the duration of EBF across the 3 groups, where we hypothesize higher rates of EBF among mothers in the conditional group.

**Results:**

Recruitment began on March 6, 2024. As of September 2025, we enrolled 204 participants. Our goal is to recruit 400 mother-infant dyads by October 2025, with study visits expected to be completed by October 2027.

**Conclusions:**

We hypothesize that the Social Transfers for Exclusive Breastfeeding in Brazil (STEBB) intervention will positively impact breastfeeding mothers in São Paulo. If successful, the program may inform national policy to enhance Brazil’s existing social transfer program for new mothers.

**Trial Registration:**

ClinicalTrials.gov NCT06157697; https://clinicaltrials.gov/study/NCT06157697

**International Registered Report Identifier (IRRID):**

DERR1-10.2196/75796

## Introduction

### Background

Breastfeeding is one of the most cost-effective ways to improve health and development outcomes for infants [1]. The World Health Organization (WHO) recommends that infants be exclusively breastfed for the first 6 months of life [2]. Exclusive breastfeeding (EBF) is defined as infants receiving only breast milk, with no other liquids or solids given, with the exception of oral rehydration solution, vitamins, minerals, or medicines [2]. Infants who are exclusively breastfed for a longer duration have strengthened immune systems, lower rates of infectious diseases, improved cognitive development, and overall lower mortality rates [3-9]. Mothers who exclusively breastfeed have improved mental health outcomes and reduced rates of breast and ovarian cancer [10,11]. Although the positive impacts of EBF are recognized internationally, the Global Breastfeeding Collective’s goal of an overall EBF rate of 50% by 2025 will be hard to reach [12,13]. Globally, the most commonly documented barriers to EBF are financial constraints, time commitment, insufficient maternity protection, and a lack of breastfeeding support [14-16]. In the last decade, many countries have launched public health campaigns, such as educational awareness programs, to improve rates of EBF. Awareness programs are important but often do not take into account the financial and time constraints associated with breastfeeding and are not always adapted for mothers from lower socioeconomic communities [17-22].

Social transfer programs have been effective in improving health outcomes while also reducing socioeconomic inequalities, especially in low- and middle-income countries [23-25]. Puerto Rico launched a small-scale cash transfer program and found an increase in breastfeeding outcomes for those receiving a financial incentive [26]. In addition, a recent study conducted in Vientiane, Lao People’s Democratic Republic (LPDR), demonstrated that a social transfer program significantly improved EBF rates at 6 months postpartum [27]. Mothers who received a conditional social transfer had the highest prevalence of EBF at 6 months, followed by those who received an unconditional social transfer [27].

Brazil has one of the highest levels of economic and health inequalities globally [28]. Most of Brazil’s wealth is held by 1% of the population, demonstrating the extreme disparities faced in the country [29,30]. To reduce these prominent wealth and health gaps, Brazil implemented a national conditional cash transfer program called Bolsa Família in 2003, which provides financial incentives to families from low-income communities to help support and improve child health and education [31,32]. Although Bolsa Família has been associated with reductions in poverty and improvements in child health outcomes, disparities continue to persist. These differences are also reflected in breastfeeding practices, where low-income mothers have lower rates of EBF compared to mothers with high income [33,34]. Brazil reports an overall EBF rate of 39% for children aged below 6 months [35,36]. While EBF rates are 46% in high-income communities, they are only 33% in low-income communities [37-39]. To address this, Auxílo Nutriz was introduced within Bolsa Família, offering an additional social transfer of 50 Brazilian Reais (US $10) to support new mothers during the first 6 months postpartum. This program is still in its early stages; therefore, data on outcomes have yet to be evaluated.

### This Study

This protocol aims to implement a randomized controlled trial (RCT), the Social Transfers for Exclusive Breastfeeding in Brazil (STEBB) program in São Paulo, to assess the effectiveness of a targeted social transfer intervention in an upper-middle-income country with persistent inequities. Building upon the design of a successful program conducted in LPDR, the STEBB intervention will evaluate the impact of breastfeeding education combined with either no social transfer, an unconditional transfer, or a conditional transfer on EBF outcomes. By leveraging Brazil’s existing national social transfer program, this study will explore whether a more focused and enhanced intervention can further promote EBF. The primary outcomes of this study are the prevalence of EBF at 6 months postpartum and the overall duration of EBF. We hypothesize that mothers receiving a conditional social transfer will have the highest rates of EBF at 6 months and, on average, the longest duration of EBF, followed by those receiving an unconditional social transfer and no social transfer.

## Methods

### Overview

The STEBB study is a prospective, 3-arm RCT conducted in São Paulo, Brazil. Before implementing the intervention, we conducted a qualitative study to identify culturally relevant social transfers and ensure social acceptability of the program.

The qualitative study involved 3 focus group discussions with mothers and fathers as well as key informant interviews with 3 health care workers and 2 representatives from the Ministry of Health and the Ministry of Social Development and Fight Against Hunger. These focus group discussions and key informant interviews assessed behaviors, perceptions, and barriers related to breastfeeding as well as the feasibility of implementing a social transfer program aimed at improving EBF rates. Findings from the qualitative research informed the design of a culturally appropriate and acceptable social transfer intervention for participants in Brazil, including options such as diapers, hygiene products, children’s clothes, toys, or books.

### Recruitment

**Figure 1:**
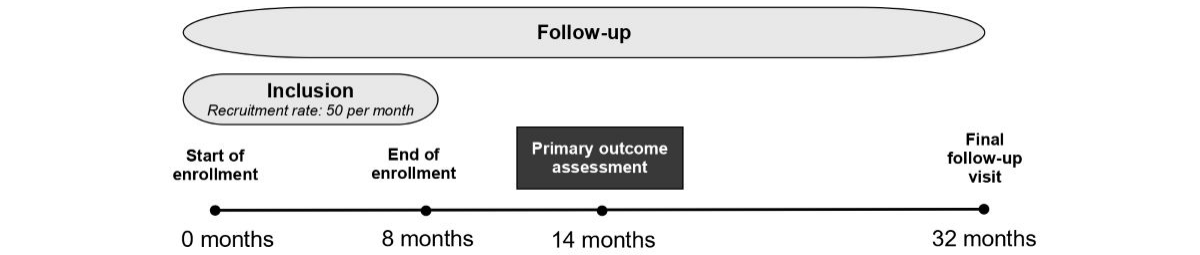
Study timeline.

A total of 400 mother-infant dyads will be recruited at the University Hospital of São Paulo and Amparo Maternal during the postpartum hospitalization period. Recruitment will take place over 8 months, with a target of enrolling 50 dyads per month. Each dyad will be followed for a period of 2 years (Figure 1).

**Figure 2:**
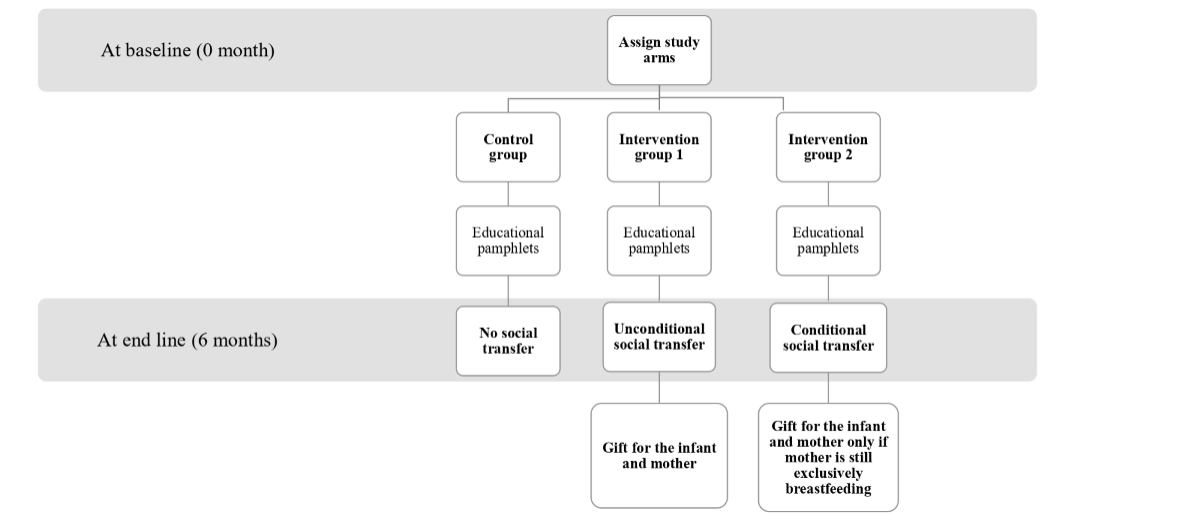
Study arms.

At baseline, mothers will be randomly assigned to one of the three groups (Figure 2): (1) control group—no intervention; (2) intervention arm 1—receipt of a social transfer at the 6-month visit; and (3) intervention arm 2—receipt of a social transfer at the 6-month visit, conditional on EBF status at the 6-month visit. All 3 groups will receive educational material on breastfeeding. The intervention is structured into 3 study arms to enable comparison between the 2 intervention groups and the control group, allowing us to evaluate the effects of conditional versus unconditional programs. Evidence from LPDR has shown that conditional social transfers significantly improved EBF rates [27]. Bolsa Família is another example of a successful conditional social transfer program for child health outcomes [34]. By contrast, our qualitative study found that participants expressed concerns about conditional programs targeting breastfeeding outcomes, particularly due to the pressure placed on mothers and the lack of reliable tools to accurately assess EBF. Given this context, we aim to evaluate both types of interventions to determine their respective impacts and assess which intervention is more effective and feasible for potential large-scale implementation.

The study participants will be contacted at the hospital after giving birth. New mothers will be eligible to participate in the study if they (1) gave birth in the last 72 hours, (2) are exclusively breastfeeding at the time of recruitment and intend to continue to exclusively breastfeed, (3) are beneficiaries of the Bolsa Família program or part of the eligible population for Bolsa Família registered in the Unified Social Assistance Registry (Cadastro Único), (4) live in São Paulo, (5) have no illnesses that contraindicates breastfeeding, (6) had a healthy singleton infant of 37 weeks or more gestation with a birth weight of at least 2500 g, and (7) agree to participate and sign an informed consent form (ICF). If underage (aged between 12 and 17 years), a legal representative will also have to agree to sign the informed consent.

A team of 2 nurses will be responsible for enrollment and follow-up of participants. The nurses will explain the study to all participants and collect the written ICF. Randomization will be done using a simple random number draw generated by ODK, used to collect data on tablets. The nurses will not have access to the random allocation sequence.

### Sample Size and Power Calculation

We initially planned to only recruit mother-infant dyads at the University Hospital of São Paulo based on the documented average of 200 births per month in 2024. However, after 2 months of recruitment, we observed a lower-than-expected birth rate. To ensure timely enrollment, we expanded our recruitment to include a second recruitment center, Amparo Maternal, which reported an average of 400 births per month in 2024. As a multicenter study, we are confident in reaching our target sample size of 400 dyads by October 2025.

We plan to enroll 200 participants in the control arm and 100 participants in each of the 2 intervention arms. This sample size provides 80% power to detect an increase in EBF from an anticipated 32.5% (65/200) in the control arm to 47% (94/200) in either intervention arm.

### Study Design

**Figure 3:**
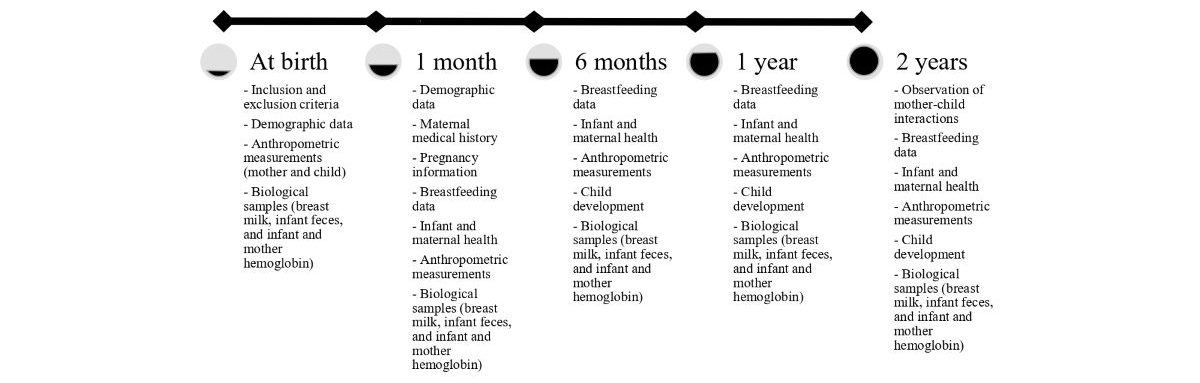
Study visits and data collection.

This study includes 5 visits: at birth (baseline), 1 month, 6 months (end line), 1 year, and 2 years (Figure 3). At baseline, the participants will complete a short questionnaire, and biological samples will be collected from both the mother and infant. At the end of the visit, the nurse will provide detailed information on the intervention depending on the group the mothers are randomly assigned to. If the mothers are assigned to an intervention group, they will be given a gift catalog to choose a social transfer for the visit at 6 months. The catalog will offer gift packages ranging from baby clothes, hygiene products, transportation vouchers, and developmental toys for the child. All 3 groups will receive educational pamphlets explaining the benefits of breastfeeding and providing breastfeeding guidelines.

During the follow-up visits (at 1 month, 6 months, 1 year, and 2 years), data collection will include a questionnaire and biological samples from both the mother and infant. The intervention groups (unconditional and conditional) will receive a social transfer at the 6-month visit. For mothers in the conditional group, assessors will review the maternal feeding reports as well as visually verify milk expression during biological collection.

### Data Collection Tools

STEBB provides a questionnaire at each visit.

#### Baseline: At Birth

The baseline questionnaire is given to the mothers during postpartum hospitalization. The questionnaire will consist of the inclusion criteria and basic demographic questions, such as education, employment, and marital status. We will also extract anthropometric measurement data from the child (weight, length, mid and upper arm circumference, abdominal circumference, chest circumference, and head circumference) and mother (blood pressure, height, weight, and heart rate) through medical records. Hemoglobin levels of the mother and infant will be collected and measured using the HemoCue Hb 301. Infant feces and human milk samples will also be collected.

#### Follow-Up: 1 Month Postpartum

The 1-month questionnaire is given to the mothers at 1 month postpartum during a hospital follow-up visit. The questionnaire includes maternal medical and obstetric history; child health (infectious and chronic illnesses, hospital visits, medication, vitamins, and vaccinations); anthropometric measurements from mother (height and weight) and child (weight, length, mid and upper arm circumference, abdominal circumference, chest circumference, and head circumference); breastfeeding and infant feeding; Resilience Scale-14 [40]; Breastfeeding Self-Efficacy Scale–short form [41]; Perceived Maternal Parenting Self-Efficacy Scale [42]; Iowa Infant Feeding Attitude Scale [43]; Postpartum Specific Anxiety Scale [44]; Edinburgh Postnatal Depression Scale [45]; Growth, Resilience, Integrity, and Tenacity Scale–short form [46]; household assets; family and gender roles; and maternal intimacy and behavioral measurements (diet, tobacco use, and alcohol consumption). This information will be collected during the 1-month visit. Mother and infant hemoglobin levels, along with infant feces and milk samples, will also be collected.

#### End line: 6 Months Postpartum

The end line questionnaire is given to the mothers at approximately 6 months (1 week earlier or later) postpartum during an at-home visit. The questionnaire includes child health (as done at 1 month), maternal and child anthropometric measurements (as done at 1 month), breastfeeding and infant feeding, Big Five Personality Scale [47], Iowa Infant Feeding Attitude Scale, Perceived Stress Scale [48], Postpartum Specific Anxiety Scale, Edinburgh Postnatal Depression Scale, Caregiver Reported Early Development Instruments–short form [49], and maternal intimacy and behavioral measurements (diet, tobacco use, and alcohol consumption). Mother and infant hemoglobin levels, along with infant feces and human milk samples, are also collected.

#### Follow-Up: 1- and 2- Years Postpartum

Additional follow-up visits will occur at 1 and 2 years postpartum (1 month earlier or later) at the participants’ homes. The questionnaire includes maternal and child health and anthropometric measurements (as done at 1 month), breastfeeding and infant feeding, Caregiver Reported Early Development Instruments–long form [49], Me As A Parent Scale [50], Perceived Stress Scale, Brief Infant Sleep Questionnaire–short form [51], maternal intimacy and behavioral measurements (diet, tobacco use, and alcohol consumption), and other questions about daycare, screen time, baby-led weaning, and parenting styles. At 2 years postpartum, child development will also be assessed using the Global Scale for Early Development–long form [52]. Mother and infant hemoglobin levels, along with infant feces and human milk samples, will also be collected.

### Outcomes

The primary study outcome is the prevalence of EBF at 6 months postpartum as well as EBF duration. The secondary outcomes will include complementary breastfeeding duration and child development. Bile acids and other human milk components will be evaluated to determine the relationship with infant gut microbiota and infant growth and development.

### Statistical Analysis

All participants enrolled in this study will be included in the analysis and will be assessed by intervention group (control, unconditional, and conditional). EBF duration will be analyzed after completion of end line visits using an adjusted Cox proportional hazards model to estimate hazard ratios and 95% CI limits. We will use an adjusted logistic regression model to assess EBF at 6 months in the intervention and control groups. A complete case analysis will be used to address missing data.

### Data Management

ODK software will be used to collect and manage the study data. Questionnaires are uploaded onto the ODK platform before data collection, allowing study staff to access the questionnaire offline using a tablet or smartphone. All data collection devices are password protected and stored securely at the hospital when they are not in use. Once the questionnaires are filled out, they are uploaded onto the server and are accessible through a secure web interface, ODK Aggregate. Only authorized personnel who require the data to fulfill their duties within the scope of the research project will be able to access ODK Aggregate.

The collected data will be stored on the secure ODK server and will only be identifiable by a unique participant number. The biological specimen samples will only be identifiable by the same unique participant number. The biological specimens will be stored securely at the University of São Paulo and will only be accessible to authorized personnel. Health-related data will be stored for 15 years, following the University of São Paulo regulations. If participants sign a separate consent form for reusing health data, data and biological samples will be kept for an unspecified amount of time.

### Ethical Considerations

This study was submitted and approved by the National Commission for Ethics in Research and the University Hospital of the University of São Paulo in Brazil (6.704.768), and it was submitted for ethical review to the Ethical Commission of Northwestern and Central Switzerland (AO_2024-00015). The study will be carried out in accordance with the Declaration of Helsinki. Any amendments to the protocol, ICF, or any other forms related to the study have to be reviewed by an independent ethics committee.

Eligible participants will be identified by study staff, and an initial visit will occur at the hospital. The nature of the study will be explained to the participants orally before obtaining a written ICF. Once the participants have some time for reflection and to answer any questions, they will be asked to sign an ICF according to their assigned group. For minors (aged <18 years), both the mother and her parents will be required to provide consent. For participants who are illiterate, an additional witness will be required. Participation is voluntary, and participants have the right to withdraw from the study at any given point in time with no further obligations. If the participant decides to withdraw from the study at any time, they may request that their data will be deleted and their biological specimen samples destroyed.

Project data and biological materials will be handled with the utmost discretion and will be accessible only to authorized personnel who require the information to perform their duties within the scope of the research project. Participants will be identified solely by a unique participant number and participants will be assured that all personal information collected during the research study will be kept confidential. Given that the intervention is primarily designed to support mothers, adverse outcomes seem unlikely. Breast milk samples will be collected through hand expression, which should not be painful for participants. For the hemoglobin measurements, blood will be collected through a finger prick, which can lead to a small discomfort for the participants. The risk of inflammation or infection is small if the participants follow proper hygienic procedures. If the participant feels any discomfort, the process will be stopped. Breastfeeding mothers may experience mastitis, which may affect breastfeeding. We will provide information and advice in the educational pamphlets on how to treat the infection without having to stop breastfeeding, as recommended by the WHO.

A social transfer will be provided to participants in the intervention groups. Four gift package options will be given to allow individualized social transfers. If any negative themes arise (eg, stigma), strategies to overcome these will be identified during the qualitative study. In the case that mothers involuntarily stop breastfeeding, a small gift will be provided.

### Trial Registration

The RCT was registered on ClinicalTrial.gov on December 6^th^ 2023 (NCT06157697).

## Results

Recruitment for the study began on March 6, 2024. As of September 2025, we have enrolled 204 participants. Our goal is to recruit 400 mother-infant dyads by October 2025, with our primary outcome to be completed by May 2026 and all study visits expected to be completed by October 2027.

## Discussion

### Anticipated Findings

The STEBB intervention will advance our understanding of the main barriers to breastfeeding in an upper-middle-income country while assessing the effectiveness of a social transfer intervention for increasing EBF rates. The anticipated main finding of this study is that mothers receiving a social transfer, whether conditional or unconditional, will show improved EBF outcomes in São Paulo, Brazil. We believe that providing incentives through a social transfer program targeted at breastfeeding mothers from low-income communities offers a unique opportunity to reduce economic and health inequalities while simultaneously increasing EBF rates.

The STEBB intervention is one of the first social transfer programs implemented in an upper-middle-income country, specifically targeting EBF outcomes. It builds on a social transfer program implemented in LPDR in 2022, which was the first study to demonstrate that both conditional and unconditional social transfers can improve EBF outcomes [27,53]. Capitalizing on the ongoing program in LPDR and insights from our qualitative research, the STEBB intervention was adapted by applying lessons learned from the field and adjusting them to different country contexts.

While other social transfer programs have been implemented in the past, most aim to improve health and educational outcomes. Examples from South America include Bolsa Família in Brazil, Prospera in Mexico, and Más Familias en Acción in Colombia [54]. These programs have been successful in providing financial support to vulnerable populations and improving key development indicators [32,54-56].

If STEBB is successful in improving rates of EBF in São Paulo, we plan to extend the RCT to other parts of Brazil. We also see potential for informing the national social transfer program, the Bolsa Família and Auxílio Nutriz, on possibilities to improve the program outcomes, specifically regarding EBF. For future research, we plan to reproduce the intervention program in other countries and regions, particularly those with low EBF rates and where large health gaps are present.

Our dissemination plan includes sharing and discussing study findings with the local community in São Paulo, including the University Hospital of São Paulo and Amparo Maternal, as well as with policymakers in Brazil. We also plan to present our results at both local and international conferences and through publications in peer-reviewed journals.

### Limitations

One limitation for STEBB is that the social transfers provided appear relatively small. Due to ethical regulations in Brazil, we are unable to offer financial compensation for the mothers’ work time. Many low-income mothers in São Paulo are considered informal workers and are unable to take paid time off from work or do not have a safe place to pump. Because of the additional barriers low-income mothers face, breastfeeding is difficult, leading to low rates of EBF in these communities. The qualitative study that was conducted before implementation identified ways to overcome these barriers and reduce any additional burdens that these working mothers may face while breastfeeding.

## Appendix

Multimedia Appendix 1 Peer-review report from the Swiss National Science Foundation.

## Abbreviations

EBF: exclusive breastfeeding
ICF: informed consent form
LPDR: Lao People’s Democratic Republic
RCT: randomized controlled trial
STEBB: Social Transfers for Exclusive Breastfeeding in Brazil
WHO: World Health Organization

## Data Availability

The datasets generated or analyzed during this study are not publicly available due to ongoing grant analyses that have not been completed but are available from the corresponding author on reasonable request.
